# The Role of the Surface Nano-Roughness on the Wettability Performance of Microstructured Metallic Surface Using Direct Laser Interference Patterning

**DOI:** 10.3390/ma12172737

**Published:** 2019-08-27

**Authors:** Alfredo I. Aguilar-Morales, Sabri Alamri, Bogdan Voisiat, Tim Kunze, Andrés F. Lasagni

**Affiliations:** 1 Fraunhofer Institute for Material and Beam Technology IWS, Winterbergstrasse 28, 01277 Dresden, Germany; 2 Institute for Manufacturing Technology, Technische Universität Dresden, George-Baehr-Str. 3c, 01069 Dresden, Germany

**Keywords:** direct laser interference patterning, nanosecond and picosecond laser pulses, multi-scale microstructures, wettability transition

## Abstract

Superhydrophobic natural surfaces usually have multiple levels of structure hierarchy, particularly microstructures covered with nano-roughness. The multi-scale nature of such a surface reduces the wetting of water and oils, and supports self-cleaning properties. In this work, in order to broaden our understanding of the wetting properties of technical surfaces, biomimetic surface patterns were fabricated on stainless steel with single and multi-scale periodic structures using direct laser interference patterning (DLIP). Micropillars with a spatial period of 5.5 µm and a structural depth of 4.2 µm were fabricated and covered by a sub-micro roughness by using ultrashort laser pulses, thus obtaining a hierarchical geometry. In order to distinguish the influence of the different features on the wettability behavior, a nanosecond laser source was used to melt the nano-roughness, and thus to obtain single-scale patterns. Then, a systematic comparison between the single- and multi-scale structures was performed. Although, the treated surfaces showed hydrophilic behavior directly after the laser treatment, over time they reached a steady-state hydrophobic condition. However, the multi-scale structured metal showed a contact angle 31° higher than the single-scale geometry when the steady-state conditions were reached. Furthermore, the impact of the surface chemistry was investigated by energy dispersive X-ray spectroscopy (EDX) and X-ray photoelectron spectroscopy (XPS) analyses. Finally, a hydrophobizing agent was applied to the laser treated samples in order to further enhance the water contact angles and to determine the pure contribution of the surface topography. In the latter case, the multi-scale periodic microstructures reached static contact angles of 152° ± 2° and a contact angle hysteresis of only 4° ± 2°, while the single-scale structures did not show superhydrophobic behavior. These results definitely suggest that multi-scale DLIP structures in conjunction with a surface chemistry modification can promote a superhydrophobic regime.

## 1. Introduction

The enhancement of surface properties is currently addressed by using biomimetics since biological species represent optimized solutions to particular environmental conditions [1]. In particular, wetting control has been extensively investigated due to several potential applications that benefit from this property; these range from drag reduction over anti-icing to self-cleaning [2,3]. Superhydrophobic behavior, that is, the capacity to repel water, is typically achieved if the static water contact angle (SWCA) exceeds 150°. Conventionally, (super)hydrophobic surfaces can be produced by three methods: (i) by chemical modification of the surface with the help of low surface free energy materials [4,5,6]; (ii) by creating a rough topography on a surface, typically in the micrometer/nanometer scale [7], and (iii) by combining both approaches [8]. The main association between surface roughness, chemistry and water-repellency has already been explained by Wenzel [9], as well as by Cassie and Baxter [10]. Briefly, the Wenzel model describes a wetting regime in which the liquid penetrates into the roughened surface (complete contact between liquid and solid surface is always present), whereas the Cassie-Baxter model outlines the importance of trapped air between the solid surface and liquid [11]. In addition, the contact angle hysteresis (CAH) also has to be considered, which is defined as the difference between advancing and receding contact angles (also known as the dynamic contact angle). For surfaces exhibiting a Wenzel behavior, the CAH is typically high while in the case of the Cassie-Baxter condition it assumes low values. Therefore, superhydrophobic surfaces should have a SWCA above 150° as well as CAHs below 10° [12].

From a biomimetic point of view, leaves can serve as models for bio-inspired surfaces and enable us to understand the mechanisms related to the wettability of solid surfaces [13]. Natural surfaces exhibit diverse morphologies [14,15,16] with combinations of micro and nanostructures as the basis of their water repellency. In general, the surface morphology observed on leaves is subdivided into a primary topography, with feature sizes in the range of 20 to 50 µm, and a secondary one associated with an epicuticle wax layer, for instance, on the lotus leaf with sizes around 0.3 – 1.0 µm and diameters of ~ 80–120 nm [17,18]. Thus, these surfaces are characterized by at least two-level surface structures, which typically have pillar-like features in the microscale and hair-like structures in the nanoscale.

Experimental studies carried out on the lotus leaf have shown the importance of the nanoscale hair-like structures, which were responsible for an increase in the contact angle from 126° to 142° [19]. However, artificial methods to create superhydrophobic surfaces have been inspired not only by plants but also by other natural surfaces [20]. For example, the multi-scale structures observed in the comb-like patterns on the springtail skin [21] and the dual-biomimetic shark skin [22] also have a superhydrophobic surface. Therefore, it can be concluded that in general, higher contact angles are achieved on multi-scale microstructures compared to those with single-scale roughness. Additionally, a wettability state designated as the rose petal effect can be seen on hierarchical surfaces [20,23], this is correlated with a high level of adhesion between the surface and the droplet, which leads to high SWCA and high CAH values due to a mixed-state wetting regime. In this regime, a high CAH is measured if the water penetrates into the microstructure but not into the nanostructures, and therefore, high solid-water adhesion occurs [24].

An effective laser-based method to fabricate periodic patterns in the micro- and sub-micrometer range is direct laser interference patterning (DLIP). The DLIP technology is based on the interference patterns that are produced when two or more coherent laser beams are overlapped, creating a periodic variation in the laser intensity distribution. The geometry of the produced pattern is determined by the number of laser beams, the light polarization, laser energy and incident angles of each beam. These periodic patterns can be directly transferred onto the surface of different materials, and offer a wide range of topographies including line- pillar- and dot-like patterns, among others [25,26,27]. DLIP-treated surfaces have performed promisingly in diverse fields of application including the enhancement of surface properties on metals and polymers related with wettability [28,29,30], friction reduction [31] and cell adhesion [32,33,34]. An advantage of the DLIP technology is that multi-scale periodic micro and nanostructures can be produced in a single fabrication step with high throughputs and repeatability [35]. In the case of multi-scale structures, the DLIP features are covered by so-called laser induced periodic surface structures (LIPSS) formed by the interference effects of the incident laser radiation with surface electromagnetic waves, which are initiated by surface plasmon-polariton waves on metals when ultrashort laser sources are used [36].

In this study, single-scale and multi-scale periodic micro/nanostructured surfaces were produced on stainless steel. The main objective was to elucidate the contribution of the different length scales to the water contact angle. The microstructured surfaces were produced using picosecond-DLIP (ps-DLIP), where LIPSS were also obtained simultaneously. For removing the small features from the pillar-like topography, a pulsed nanosecond laser source was used, which allows melting these features, and thus obtaining single-scale patterns. The chemical composition of the laser-treated surfaces was analyzed by Energy Dispersive X-ray spectroscopy (EDX) and X-ray photon spectroscopy (XPS) measurements. To differentiate the contribution of the surface topography and chemistry to the water contact angle (WCA), a hydrophobizing agent was also applied on the different fabricated patterns. The topography of the produced patterns was analyzed using scanning electron microscopy (SEM), confocal microscopy (CM) as well as Fourier transforms to retrieve the different length scales.

## 2. Materials and Methods

### 2.1. Materials

Electro-polished metal sheets of corrosion resistant ferritic steel X6Cr17 (EN 1.4016) (composition in % by mass: C of 0.08, Si of 1, Mn of 1, P of 0.040, Cr of 16–18, S ≤ 0.015), and 0.78 mm thick were used as substrates. As-received, the samples have an initial roughness Ra of 60 nm. Prior to the laser process, the substrates were cleaned of contamination using ethanol. After the laser process, the samples were not subject to any further cleaning procedures.

### 2.2. Picosecond Direct Laser Interference Patterning Setup

**Figure 1:**
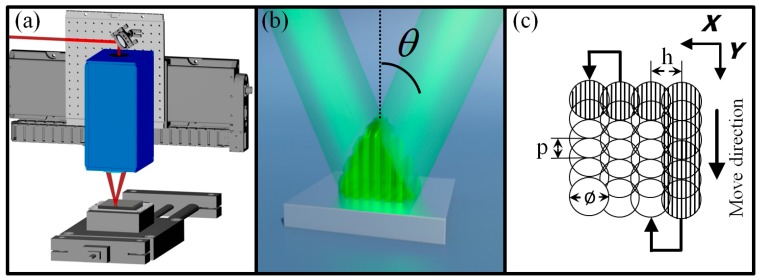
(**a**) Schema of the used two-beam direct laser interference patterning (DLIP) setup including the DLIP module and the translational stages; (**b**) Representation of a two-beam interference pattern at the materials surface; (**c**) Strategy utilized to process the samples: ø, h and p denote the diameter of the interference area, the hatch distance and the pulse-to-pulse overlap, respectively.

The metallic substrates were structured using a two-beam DLIP configuration, which results in the generation of line-like patterns. The experimental DLIP setup (represented in Figure 1a) includes an infrared (IR) pico-second laser emitting 10 ps pulses at the wavelength of 1064 nm (solid-state Q-switched Innoslab Nd:YVO4) (EdgeWave, Würselen, Germany), operating at a repetition rate of 1 kHz with a TEM00 beam intensity distribution with M^2^ < 1.1. The beam is horizontally polarized. The DLIP optical module (developed at Fraunhofer IWS) allows the main laser beam to be split into two sub-beams, which are overlapped on the sample surface with a characteristic angle θ (see Figure 1b); this is automatically adjustable in the optical module. The angle θ together with the laser wavelength λ determines the spatial period Λ of the pattern, which is defined as the repetitive distance between two intensity maxima or minima (Λ = λ/(2 sin θ))[27]. Additional information about the developed optical DLIP heads has already been published elsewhere [37].

For these experiments, a spatial period of 5.5 µm was used. The diameter ø of the area, in which the interference pattern is produced (DLIP-pixel), was set to 340 µm. The pulse energy measured was 0.67 mJ, which results in a laser fluence of 0.74 J/cm^2^; this value was kept as a constant during the experiments. To extend the size of the treated area, the sample was moved in the *x* and *y* directions according to the processing strategy depicted in Figure 1c. The overlap of pulses was performed in the *y*-direction (move direction) with a pulse-to-pulse overlap (p) of 99% and a linear speed of 0.34 cm/s. Note that the DLIP pattern is oriented parallel to the moving direction (and perpendicular to the beam polarization). In the *x*-direction, the pulses were separated by a hatch distance (h) of 308 µm in order to ensure homogeneous coverage of the processed area and a perfect overlap between the interference maxima of two hatched DLIP-pixels. For the fabrication of pillar-like microstructures, the areas containing line-like patterns were re-irradiated after rotating the substrate by 90° (*x-y* plane) and repeating the procedure described above. All laser experiments were carried out under normal ambient-pressure conditions.

### 2.3. Nanosecond Direct Laser Treatment Setup

To melt the LIPSS features (which were fabricated simultaneously during the DLIP process using picosecond laser pulses), and thus obtain the single-scale patterns with comparable structure height, the samples were processed using a 12 ns pulsed Q-switched Nd:YLF laser (TECH-1053 Basic) (Laser-Export, Moscow, Rusia). The laser wavelength was 1053 nm and a repetition rate of 1 kHz was used. The laser provided a TEM00 beam intensity distribution with a M^2^ < 1.2 and the spot size at the working position had a diameter of 145 μm. To cover larger areas, the substrates were translated in *x* and *y* directions following the same strategy depicted in Figure 1c with a pulse-to-pulse overlap of 90% and a hatch distance of 75 µm. The laser fluence was varied between 0.19 J/cm^2^ and 0.55 J/cm^2^.

### 2.4. Surface Chemical Process

Samples were chemically treated by dipping them in a hydrophobizing agent (Mecasurf^®^) (Surfactis, Angers, France) after the laser processes. This agent (ethoxydifluoromethyl, C_13_H_3_F_27_O_2_) used covalently bonded fluoride groups to cover the surface, saturating the material with non-polar compounds (reducing the surface energy) with an ultrathin layer (< 5 nm). It is important to mention, that the chemical reaction can take place only if the surface is not passivated with organic compounds. Therefore, the samples must be chemically treated directly after the laser process. The dipping process consists of immersing the samples for five minutes in the hydrophobizing agent. Afterwards, the sample was removed left for about two minutes until the solvent was completely evaporated.

### 2.5. Surface Characterization

The treated surfaces were characterized using confocal microscopy (LeicaSCAN DCM3D) (Leica microsystems, Wetzlar, Germany) with 150 X magnification, allowing lateral and vertical resolutions of 140 nm and 2 nm, respectively. Scanning electron microscopy (SEM) images and energy dispersive X-ray spectroscopy (EDX) analyses were carried out using a Zeiss Supra 40VP (Carl Zeiss, Oberkochen, Germany) at the operating voltage of 5 kV.

XPS measurements were carried out using an Amicus spectrometer (Kratos Analytical, Manchester, UK) equipped with a non-monochromatic Mg Kα X-ray source operated at 240 W and 8 kV. The kinetic energy of the photoelectrons was determined using an analyzer with pass energy of 75 eV. The take-off angle between sample’s surface normal and the electro optical axis of the spectrometer was 0°. In this case, the information depth is about 8 nm. The spectra were referenced to the C1s peak of aliphatic carbon at a binding energy of 285.0 eV. A satellite subtraction procedure was applied. Quantitative elemental compositions were determined from peak areas using experimentally determined sensitivity factors and the spectrometer transmission function.

SWCA and CAH were conducted in a contact angle measurement system (OCA 20,) (Dataphysics Instruments GmbH, Filderstadt, Germany) using the tangent searching fitting mode during measurements. The SWCA measurements were conducted using 4 µL deionized water. Advancing contact angles were measured by gradually increasing the volume of the water droplet from 4 µL up to 12 µL at a rate of 0.9 µL/s. For the receding mode, 8 µL were retired. The contact angles were monitored up to 180 days after the fabrication process.

## 3. Results and Discussion

### 3.1. Fabrication of Single- and Multi-Scale Microstructures Using DLIP

**Figure 2:**
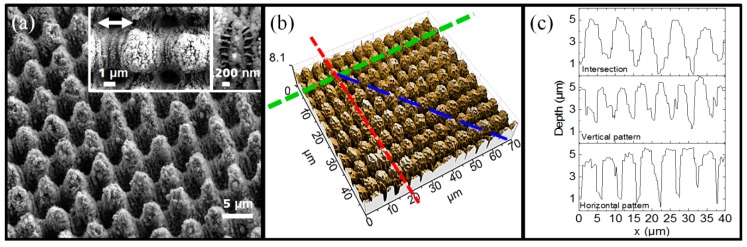
(**a**) Scanning electron micrograph (tilted at angles of 20° in *x* and 10° in *y*), the arrow denotes the beam polarization correlated with the irradiation after rotating the substrate 90°; (**b**) confocal microscopy image of DLIP pillar-like structure on stainless steel with a spatial period Λ of 5.5 µm, produced with a laser fluence of 0.74 J/cm^2^ and 99% overlap. The lines in (**b**) denote the profiles from where the depth of the pillars was measured (green horizontal, red: vertical, and blue: intersection depths); (**c**) profiles obtained by confocal microscopy.

Pillar-like microstructures were fabricated by ps-DLIP using a spatial period of 5.5 µm and pulse-to-pulse overlap of p = 99%. The typical morphology of the produced topographies can be seen in Figure 2a. The inset in Figure 2a highlights the additional nanostructures that were simultaneously produced. These small features can be identified as low spatial frequency LIPSS (LSFL) with a spatial period of approximately 800 nm. The LSFL are oriented parallel to the second line-like DLIP treatment and are also perpendicular to the laser beam polarization (the orientation of the beam polarization is represented by the arrow in Figure 2a). In addition, high spatial frequency LIPSS (HSFL) were produced (see zoomed inset in Figure 2a) with a repetitive distance of approximately 200 nm. Differently, these structures are commonly oriented parallel to the beam polarization direction (and perpendicularly to the LSFL) [38]. The LSFL and HSFL were predominantly observable between each micropillar (at the interference maxima positions).

In addition to the LSFL and HSFL, nanoparticles with a size of about 100 nm can also be found on top of the micro-pillars, which probably result from the re-deposition of the ablated material during the structuring process [39,40]. These features are comparable in size to the nano-hair structures observed on the leaves of the Nelumbo Nucifera and Colocasia esculenta [17]. However, contrary to natural surfaces, the microstructures fabricated by ps-DLIP are highly ordered and homogenously distributed.

Figure 2b illustrates the surface topography of the treated samples measured by confocal microscopy. The microstructure depth at the intersection regions of the interference maxima-maxima is 4.2 µm ± 0.6 µm (blue line) whereas the vertical (it was measured setting the green line) and horizontal (it was measured setting the red line) depths were determined to be about 3.0 µm ± 0.4 µm. The profiles at these positions are presented in Figure 2c.

**Figure 3:**
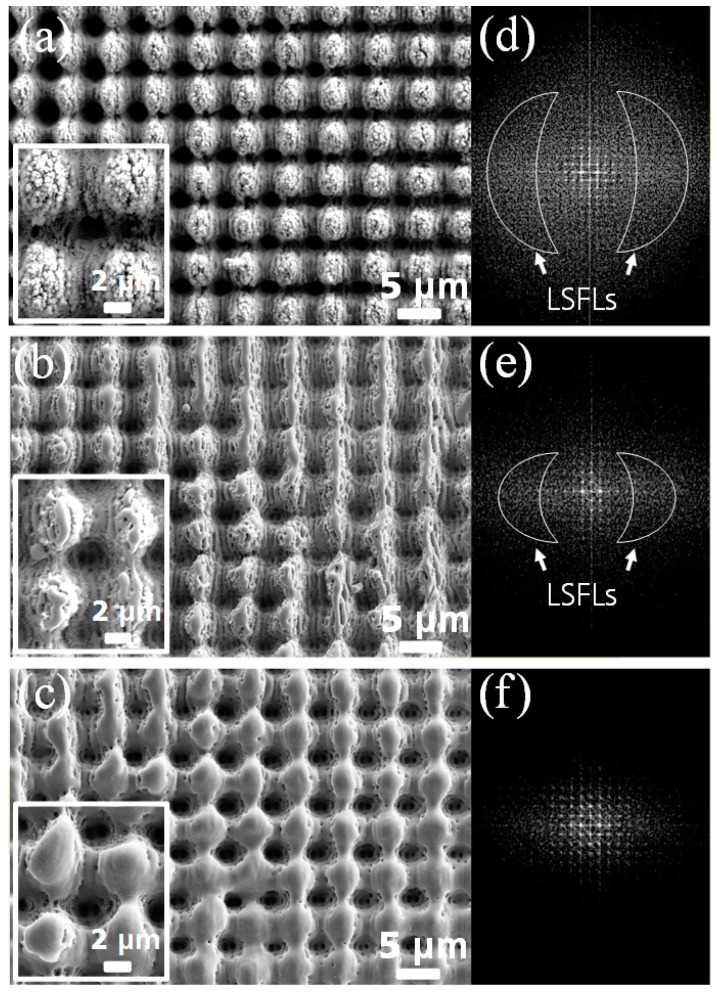
(**a**–**c**) SEM images of the stainless steel samples processed by the picosecond(ps)-DLIP technique and their 2D-fast Fourier transform (FFT) (**d**–**f**); (**a**) Multi-scale pillar-like structures covered by laser induced periodic surface structures (LIPSS) features and pillar-like structures after using the ns laser treatment for melting the LIPSS features at laser fluences of (**b,e**) 0.27 J/cm^2^ and (**c,f**) 0.55 J/cm^2^.

After producing the multi-scale surfaces using the ps-DLIP process, the LIPSS were melted using a nanosecond (ns) pulsed laser treatment. Additionally, the aim of the experiment was to keep the morphology of the pillars constant. In this way, the influence of the sub-micrometer structures on the SWCA can be properly highlighted. To qualitatively analyse this procedure, two-dimensional fast Fourier transform (FFT) was applied on the taken SEM images. The FFT analysis identifies periodic features with different length scales. In the case of the ps-DLIP treated steel sample, the SEM image is shown in Figure 3a and its Fourier transform is shown in Figure 3d. As it can be seen, the FFT image of the combined DLIP + LIPSS structures is characterized by a distinct intensity distribution with features of constant distance, which originate from the symmetry of the DLIP pattern (pillar geometry). Additionally, two flanking “clouds” are attributed to the LIPSS features, and they are classified as LSFLs type-s features [38]. This multi-scale periodic surface was later irradiated with laser fluences of 0.27 J/cm^2^ and 0.55 J/cm^2^ (see experimental section), and obtained the surface pattern topographies shown in Figure 3b,c, respectively.

As it can be seen in the images, the post-process led to the subsequent melting of the surface features (LSFL, HSFL and nanoparticles) depending on the laser fluence. In the case of the sample irradiated with a lower laser fluence (Figure 3b), the nano-roughness is reduced and at the same time the LSFL became less noticeable, which means that the nanofeatures are initially molten. Finally, a laser fluence of 0.55 J/cm^2^ leads to a nearly complete erasing of the two-scale LIPSS, as shown in Figure 3c. The continuous disappearance of the LIPSS with increasing laser fluence was also confirmed by the FFT results. As can be seen in Figure 3e,f, the intensity of the clouds corresponding to the LIPSS become less pronounced with increasing laser fluence.

**Figure 4:**
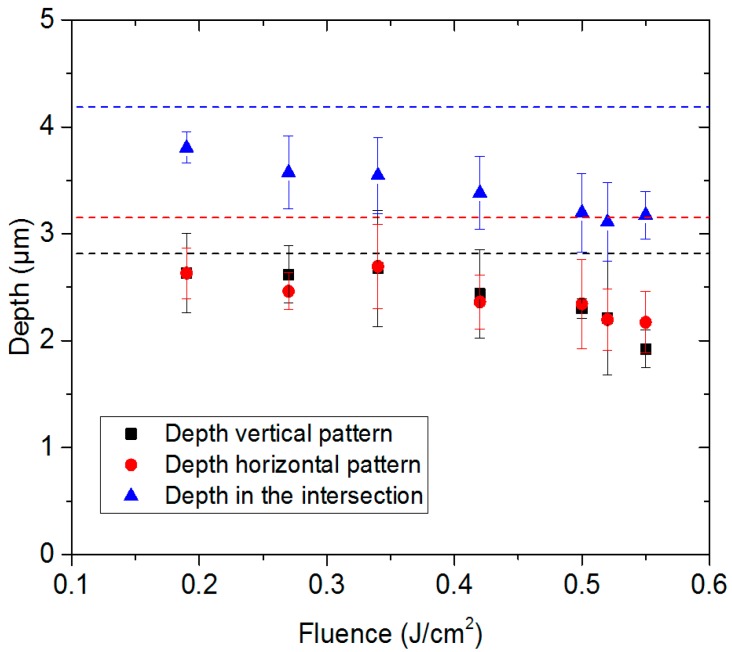
(**a**) Depth of ps-DLIP pillar-like microstructures as a function of the laser fluence used in the post-process by nanosecond laser pulses. The dashed lines denote the mean depth of the untreated multi-scale pillar-like structures at the vertical (black line), horizontal (red line) and intersection points (blue line). Each data point was an average of ten depth measurements, the error bars were estimated by the standard deviation.

Although the LIPSS could be “removed” from the produced pillars using the ps-DLIP treatment, it is also necessary to determine whether the ns pulsed treatment affected the structural depth of the pillars. This effect is depicted in Figure 4a, where the geometrical parameters of the pillars were characterized as a function of the laser fluence used during the ns pulsed laser treatment. As can be seen, the structural depth is continuously reduced with the increase in the laser fluence, from 4.2 µm ± 0.6 µm to 3.17 µm ± 0.4 µm for the untreated surface when using a laser fluence of 0.55 J/cm^2^. Note that the local heating promoted the melting of the surface at similar depth rates. In this context, it can be noted that the depth in the intersection is always larger than the depth at the horizontal and vertical pattern, because when a double exposure approach was used, this area was irradiated twice by the interference maxima position of the DLIP pattern.

### 3.2. Wettability Behavior

**Figure 5:**
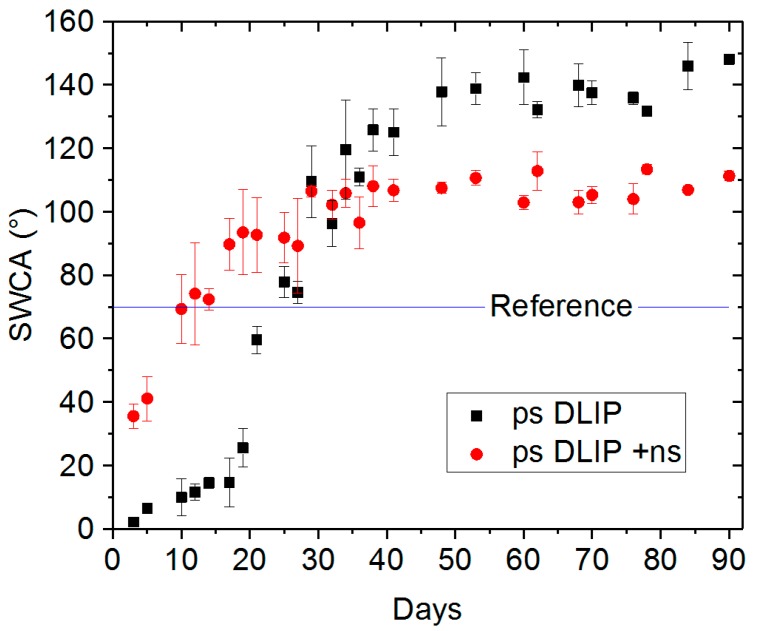
Water contact angle measurements over time of the multi-scale pillar-like structures (ps-DLIP) and single-scale patterns (ps-DLIP + ns). The spatial period Λ of the pillars was 5.5 µm. For the ps treatment, a fluence F = 0.74 J/cm^2^ and an overlap of 99% were used. Each data point is an average of four static water contact angle (SWCA) measurements, the error bars were estimated by their standard deviation. After each measurement, the samples were stored under normal ambient-pressure conditions.

In a second set of experiments, the wettability performance of both single-scale and multi-scale DLIP structures was evaluated as a function of time. The single-scale structures were obtained by applying nanosecond laser pulses with a fluence of 0.55 J/cm^2^. The long-term SWCA measurements are presented in Figure 5. As can be seen, three days after the laser irradiation, the SWCA on the multi-scale structured (ps-DLIP) surface was close to zero (3° ± 1°), while the SWCA on the single-scale pattern (ps-DLIP + ns) was 34° ± 5°.

The evolution of the SWCA over time shows two different responses, depending on the degree of complexity of the microstructures. In fact, for the multi-scale structures (ps-DLIP), the SWCA increased up to 138° ± 10° after 50 days, while for the single-scale structures (ps-DLIP + ns) values of 107° ± 2° were reached after the same period of time. Afterwards, the samples were stored under ambient conditions and the SWCA was measured each 30 days (up until 180 days) and any significant change in the wettability response was observed. Due to the observable variation in less than 20° in the SWCA measurements on each sample, it was determined that the surfaces achieved a steady-state hydrophobic condition. Note that the steady-state condition was achieved after 30 days for single-scale structures (ps-DLIP + ns), in comparison to 50 days required for the multi-scale structures (ps-DLIP). This result suggests that the nano-roughness (LIPSS features and nanoparticles) is responsible for an increase of 31° (from 107° to 138°) when the steady-state condition is reached. Although a high SWCA was reached, it is important to mention that the produced surfaces present a high CAH, 30° and 45° for the ps-DLIP and ps-DLIP + ns, respectively, which means that the water droplets can roll only after high inclination of the sample.

These observations are in agreement with a Wenzel model, for which (in a hydrophobic steady-state) an increase in the roughness induces higher contact angles, where complete wetting between the liquid and the microstructures exist. However, specifically for the multi-scale structures (ps-DLIP) values of SWCA higher than 150° and CAH higher than 30° were measured during the steady-state. These observations are connected to the rose petal effect, whereby high water droplet adhesion was promoted, and this suggested that a complete water impregnation occurred in the pillar-like microstructures while the air-trapping is maintained in the nanostructures. Less effective air trapping is associated to the single-scale structures (ps-DLIP + ns) with a CAH increase of up to 45°.

**Figure 6:**
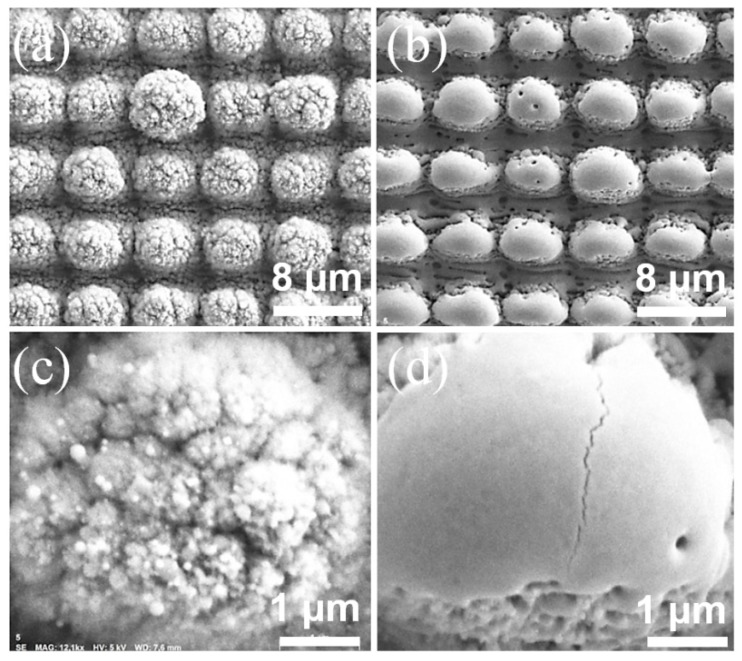
SEM images of stainless steel samples processed by ps-DLIP with pillar-like topographies, (**a**,**c**) multi-scale structures and (**b**,**d**) single-scale structures after the ns laser treatment (at 0.27 J/cm^2^). Figures (**c**) and (**d**) show a high magnification image corresponding to the top of each pillar.

However, the observed SWCA-transition has also been reported by other authors on different metallic surfaces [41,42,43,44,45,46] and it has been attributed to chemical changes at the surface topography after the laser irradiation. Therefore, in order to obtain the chemical composition of the treated surfaces, EDX analyses were conducted on both the single-scale (ps-DLIP + ns) and multi-scale structures (ps-DLIP). SEM images of the evaluated surfaces are shown in Figure 6 while the measured chemical compositions are described in Table 1. Two different measurements were carried out according to the data shown in Figure 5: (i) 7 days after the irradiation, when both samples showed a hydrophilic state, and (ii) 76 days after the laser treatment, when both samples achieved a hydrophobic state.

As it can be seen in Table 1, after 7 days an increase in the carbon content was found for the multi-scale structures (ps-DLIP). However, in case of the single-scale pattern (ps-DLIP + ns), a decrease in carbon content with respect to the untreated surface was observed. This result suggests that the ns laser treatment leads to a reduction in the carbon content.

For a more precise analysis of the chemical composition, XPS analyses were performed on the treated samples. Table 2 shows the data retrieved on the multi-scale (ps-DLIP) and the single-scale structures (ps-DLIP + ns) 2 and 53 days after the fabrication.

According to the XPS results, an increase in carbon content was measured on the treated samples 53 days after the irradiation. This highlights that the transition from the hydrophilic to the hydrophobic state is related to the decomposition of CO_2_ into carbon (accumulation of non-polar carbon compounds), which is promoted over time due to the activation of iron oxides by the laser radiation [47]. This is typically observed when irradiated metal surfaces are exposed to ambient air or a CO_2_ atmosphere [48]. The produced non-polar chemical groups on the surface reduce the wettability due to their low interaction with the polar forces and the low possibility of creating hydrogen bonds with the water molecules [49]. This reduces the surface free energy, and thus the SWCA is increased. Furthermore, it has been proposed in several works [50,51,52] that the ratio of C/Fe provides an idea of the relative amount of adsorbed non-polar carbon compounds.

In our case, the atomic ratios of C/Fe at 2 and 53 days after laser treatment increased from 3.49 to 4.16 and from 1.55 to 1.98 for multi-scale (ps-DLIP) and single-scale structures (ps-DLIP + ns), respectively. Accordingly, this means that the polarity of the surface is also reduced, resulting in higher hydrophobicity of the surface. The ratio C/O did not change (considering the measurement error is approximately +/−0.5 at. %) over time, which indicates the stable proportion of the C-O bounds.

### 3.3. Wetting Response with and without Chemical Surface Modification

**Figure 7:**
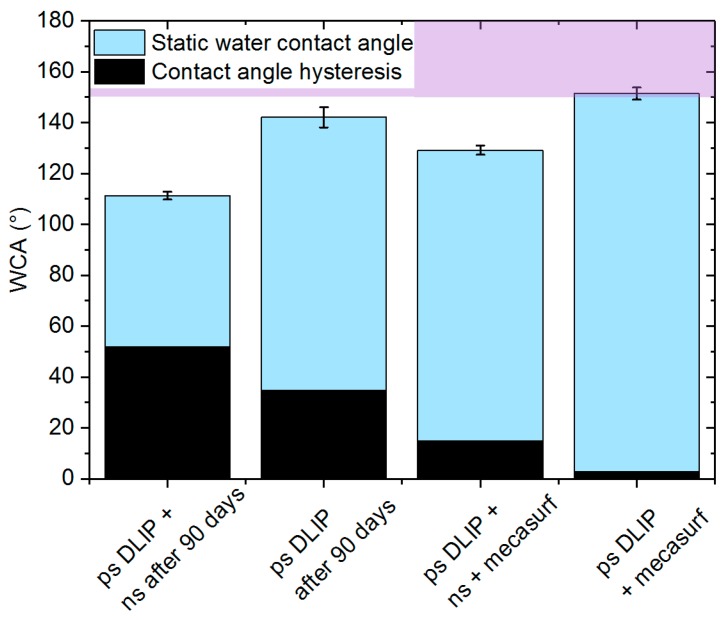
Static water contact angle (SWCA) and contact angle hysteresis (CAH) on the multi-scale (ps-DLIP) and single-scale structures (ps-DLIP + ns) measured 90 days after the laser treatment. New surfaces with the same topographies were treated with a hydrophobizing agent, permitting the multi-scale structures (ps-DLIP + mecasurf) to achieve superhydrophobic behavior. The values are an average of four SWCA measurements, the error bars were estimated by their standard deviation.

Due to the measured difference in the surface chemistry for both laser treated samples, characteristics connected with the wettability responses over the time proceeded to modify the chemistry surface. In order to provide the samples with an equal surface chemistry, new samples with single- and multi-scale topographies were fabricated and immediately after the laser treatments they were chemically treated by dipping them in a hydrophobizing agent (Mecasurf^®^) (Surfactis, Angers, France). After the chemical treatment, both SWCA and CAH angles were measured and the outcomes are shown in Figure 7. The image also includes the wettability results for the surfaces without the chemical treatment 90 days after the laser treatments (when the steady-state condition was reached).

As can be clearly observed, the superhydrophobic condition was only achieved on the multi-scale structures (ps-DLIP) covered by the hydrophobic coating (Mecasurf^®^) (Surfactis, Angers, France). In this case, the SWCA and CAH values were 152° ± 2° and 4° ± 2°, respectively. In the case of the single-scale structures (ps-DLIP + ns) also covered by the hydrophobic coating, the SWCA of 129° ± 3° was measured, that is approximately 23° lower than the multi-scale surfaces. The measured CAH was 15°, which also denotes the lower ability of the water droplets to roll over on the material surface. The results obtained for the original samples, without the Mecasurf^®^ treatment (Surfactis, Angers, France), show lower SWCAs as well as higher hysteresis values (higher CAH). However, also in this case, the multi-scale structures showed a better hydrophobic performance compared to the single-scale structures.

The results suggest that the multi-scale DLIP structures in conjunction with a surface chemistry modification promote superhydrophobic conditions. On the contrary, single-scale micro-pillars showed, independent of the surface chemistry, a poor performance compared to the multi-scaled structures, confirming the importance of hierarchical surface patterns for tailoring the wettability properties on metals.

## 4. Conclusions

Single-scale and multi-scale structures were fabricated on stainless steel, for understanding the influence of the different surface features on the SWCA and the contact angle hysteresis. The processing of metallic samples with a two-beam interference setup and a ps laser source produced multiple-scale periodic microstructures consisting of pillar-like elements combined with LSFL and HSFL features, which mimic the surface textures observed on different plants with superhydrophobic functionalities.

An approach based on the isolation of the primary microstructure produced by ps-DLIP, was developed by selectively melting the nano-texture related to LIPSS. This procedure permitted an evaluation of whether small-scale periodic nanostructures have an impact on wettability. Then, the wettability properties of the laser treated substrates were evaluated over time, showing that multi-scale microstructures reach a higher SWCA compared to single-scale structures. Moreover, the chemical analysis of the two different morphologies was compared, and an increased ratio of C/Fe on the surfaces was found 53 days after the laser treatment, which also contributed to the SWCA. Furthermore, high water droplet adhesion was identified on the multi-scale microstructures, which leads to high SWCA and high CAH; this wetting regime is correlated with the rose petal effect.

Finally, the laser processed samples were treated with a hydrophobizing agent in order to separate the contribution of the different feature size to the WCA. In this case, the multi-scale structure not only obtained the superhydrophobic condition but also presented a 31° higher SWCA than the single-scale pattern. Furthermore, the CAH was reduced to only 4°, permitting water droplets to roll easily on the laser treated surface. Consequently, it was possible to qualitatively demonstrate the positive contribution of the multi-scale structures on the superhydrophobic behaviour of stainless steel samples.

## Figures and Tables

**Table 1:** Chemical composition C, O, Fe and Cr elements (at. %) for the multi-scale structures (ps-DLIP) and single-scale (ps-DLIP + ns) treated surfaces (corresponding to Figure 6) measured by EDX.

Sample	C (at. %)	O (at. %)	Fe (at. %)	Cr (at.%)
**Untreated**	5.84	7.71	70.35	16.10
**ps-DLIP** (after 7 days)	8.10	62.43	23.75	5.72
**ps-DLIP + ns** (after 7 days)	2.24	66.88	24.85	6.03
**ps-DLIP** (after 76 days)	11.44	47.46	33.73	7.37
**ps-DLIP + ns** (after 76 days)	3.33	46.01	40.22	10.44

**Table 2:** Surface composition (at. %) of the multi-scale (ps-DLIP) and single-scale (ps-DLIP + ns) after 2 days and 53 days measured by XPS.

Sample	C 1s (at. %)	O 1s (at. %)	Fe 3p (at. %)	C/Fe	C/O
**ps-DLIP** (after 2 days)	48.66	37.42	13.92	3.49	1.30
**ps-DLIP + ns** (after 2 days)	36.78	39.57	23.65	1.55	0.92
**ps-DLIP** (after 53 days)	50.70	37.14	12.16	4.16	1.36
**ps-DLIP + ns** (after 53 days)	40.27	39.46	20.26	1.98	1.02
